# Basic and clinical immunology – 3010. The RNA-binding protein HuR coordinately regulates GATA-3 and Th2 cytokine gene expression in dose dependent manner

**DOI:** 10.1186/1939-4551-6-S1-P186

**Published:** 2013-04-23

**Authors:** Ulus Atasoy, Matt Gubin, Joseph Magee, Vincenzo Casolaro, Cristiana Stellato

**Affiliations:** 1 Surgery and Molecular Microbiology, Immunology, University of Missouri, Columbia, MO, USA; 2 Allergy and Immunology, Johns Hopkins University, Baltimore, MD, USA

## Background

Naïve CD4+ T cells can differentiate into different subsets. Whereas transcriptional regulation of CD4+ T cells is well studied, posttranscriptional control by RNA binding proteins (RBPs) and microRNAs is poorly understood. CD4+ Th2 mediated diseases such as allergen-induced asthma, are driven by GATA-3, IL-4 and IL-13. The RBP, HuR, has been shown to posttranscriptionally regulate many early response genes, including IL-4 and IL-13. GATA-3 contains AU-rich elements (ARE) in its 3’ untranslated region (UTR) which are binding sites for HuR. We first identified GATA-3, IL-4 and IL-13 as HuR targets using RIP-Chip (RNA immunoprecipitation applied to microarrays). We hypothesized that HuR may be coordinately regulating Th2 differentiation.

## Methods

We used in vitro and in vivo models, including a HuR over-expression transgenic system, as well as a tissue specific HuR conditional knock out mouse (HuR^fl/fl^) to ablate HuR in T cells. Additionally, we also used siRNA and lentiviral shRNA to knock-down HuR.

## Results

HuR over expression stabilized GATA-3, IL-4 and IL-13 mRNAs, leading to significant increases at both mRNA and protein levels for these genes. Conversely, HuR knock down using lentiviral shRNAs produced opposite results. These findings were confirmed in human lymphocytes, indicating potential clinical relevance to disease. We verified that GATA-3 is a HuR target using a combination of IP and biotin pull downs and defined HuR binding sites. Interestingly, Th2 polarized cells with reduced HuR levels (26%) from conditional HuR^fl/+^ knockout mice, had significant decreases in IL-4, IL-13 and GATA-3 mRNA but not protein. Surprisingly, CD4+ Th2 polarized cells from homozygous HuR^fl/fl^ mice with pronounced HuR knockdown (93%) showed significant increased IL-4 and IL-13 expression at both mRNA and protein levels but no changes in GATA-3 or IFN-γ. We measured both nascent mRNA transcription and stability for IL-4 and IL-13 mRNAs and found differential regulation.

## Conclusions

These data suggest there may be a critical range of HuR protein levels which regulates Th2 differentiation by interacting with different target genes. Further studies defining how RBP proteins can regulate GATA-3 and Th2 cytokine gene expression will be critical for elucidating posttranscriptional control of Th2 differentiation and of allergic lung inflammation.

